# Impact of HIV-1 genetic diversity on disease progression: a prospective cohort study in Guangxi

**DOI:** 10.3389/fcimb.2024.1415123

**Published:** 2024-06-27

**Authors:** Xianwu Pang, Jinghua Huang, Kailing Tang, Jie Ma, Ningye Fang, Haomin Xie, Qin He, Qiuying Zhu, Guanghua Lan, Shujia Liang

**Affiliations:** Guangxi Key Laboratory of Major Infectious Disease Prevention Control and Biosafety Emergency Response, Guangxi Key Laboratory of AIDS Prevention Control and Translation, Guangxi Zhuang Autonomous Region Center for Disease Control and Prevention, Nanning, China

**Keywords:** HIV-1, subtype, genetic diversity, disease progression, CD4+T lymphocyte

## Abstract

The high proportion of AIDS cases and mortality rates in Guangxi underscores the urgency to investigate the influence of HIV-1 genetic diversity on disease progression in this region. Newly diagnosed HIV-1 patients were enrolled from January 2016 to December 2021, and the follow-up work and detection of CD4+T lymphocytes were carried out every six months until December 2022. Multivariate logistic regression was used to analyze the factors affecting pre-treatment CD4+T lymphocyte counts, while local weighted regression models (LOESS) and generalized estimating equation models (GEE) were conducted to assess factors influencing CD4+T Lymphocyte Recovery. Cox regression analysis was utilized to examine the impact of subtypes on survival risk. Additionally, HIV-1 env sequences were utilized for predicting CXCR4 and CCR5 receptors. The study encompassed 1867 individuals with pol sequences and 281 with env sequences. Our findings indicate that age over 30, divorced/widowed, peasant, heterosexual infection, CRF01_AE, long-term infection, and Pre-treatment Viral load >10000 copies/ml were factors associated with higher risk for pre-treatment CD4+T lymphocyte decline. Specifically, male gender, age over 30, heterosexual infection (HETs), long-term infection, CRF01_AE, and Pre-treatment CD4 T cell counts below 350/µL were identified as risk factors impeding CD4+T lymphocyte recovery. Pre-treatment CD4+T lymphocyte counts and recovery in individuals infected with CRF01_AE were lower compared to CRF07_BC and CRF55_01B. Additionally, CRF01_AE and CRF08_BC subtypes exhibited higher mortality rates than CRF07_BC, CRF55_01B, and other subtypes. Notably, CRF01_AE demonstrated the highest percentage of CXCR4 affinity ratios. This research unveils the intricate influence of HIV-1 gene diversity on CD4+T lymphocyte dynamics and clinical outcomes. It highlights the multifaceted nature of HIV infection in Guangxi, providing novel insights into subtype-specific disease progression among HIV-infected individuals in this region.

## Introduction

HIV-1 exhibits remarkable genetic diversity, leading to distinctive coreceptor utilization and varying disease progression among its diverse subtypes and circulating strains (Poveda et al., 2006). Research underscores the differing clinical presentations and immune system impairments associated with various HIV-1 subtypes post-infection. Particularly, individuals infected with subtype D in African regions exhibit higher mortality risks and are more prone to advancing to the AIDS stage compared to those with subtype A (Vasan et al., 2006; Baeten et al., 2007; Keller et al., 2009). Studies in the United Kingdom further affirm the accelerated disease progression in subtype D-infected individuals relative to other subtypes (Easterbrook et al., 2010). Investigations focusing on the CRF01_AE subtype highlight a significantly rapid disease progression from HIV-1 infection to clinical AIDS stage and a drop in CD4+ T cell count below 200/µL, with median times of 7.2 years and 6.5 years, respectively (Rangsin et al., 2007). Moreover, studies have noted a higher prevalence of CXCR4 tropism within CRF01_AE subtypes, correlating with rapid disease progression (Utaipat et al., 2002). Additionally, substantial variations exist among distinct CRF01_AE epidemic clusters (Song et al., 2019). Similarly, among patients with the CRF02_AG subtype, studies have reported an 86% usage rate of CXCR4 tropism, associated with rapid disease progression (Esbjornsson et al., 2010).

However, the tropism observed in recombinant epidemic strains adds complexity. Coreceptor selection, dependent on HIV’s affinity for coreceptors, categorizes strains as R5-dependent (CCR5 receptor), X4-dependent (CXCR4 receptor), or mixed tropic (R5X4) capable of utilizing both receptors (Berger et al., 1998; Huang et al., 2007; Irlbeck et al., 2008). This selection significantly influences disease progression, with early-stage HIV-1 infections predominantly exhibiting R5 tropic strains (Schuitemaker et al., 1991). Yet, prolonged infection often leads to the emergence of X4-tropic or R5X4-tropic strains in some patients, associated with rapid CD4+ T lymphocyte decline and exacerbated immune function deterioration due to their ability to utilize various host cells for replication and induce stronger apoptotic effects (Koot et al., 1993; Yamashita et al., 1994).

Guangxi, a southwestern Chinese province bordering Vietnam, faces a severe HIV-1 epidemic, observing a relatively high proportion of newly diagnosed HIV-1 infected individuals diagnosed as AIDS (Chen et al., 2019; Sun et al., 2020). However, it remains unclear whether the increased proportion of AIDS patients arises from late detection or other contributing factors. Currently, prevalent HIV-1 subtypes in Guangxi include CRF01_AE, CRF07_BC, CRF08_BC, and CRF55_01B. The potential association between rapid disease progression and HIV-1 subtypes necessitates further investigation. This study aims to encompass newly diagnosed HIV-1 infected individuals in a prospective study, intending to investigate the impact of subtypes on CD4+ T lymphocyte count and mortality.

## Materials and methods

### Study participants and sample collection

Between January 2016 and December 2021, participants were recruited from Voluntary Counseling and Testing Centers (VCT) and Non-Governmental Organizations (NGO) in Guangxi. Inclusion criteria were as follows: 1) newly diagnosed with HIV-1; 2) not initiated on Antiretroviral Therapy (ART); 3) aged ≥ 18 years and provided informed consent. Peripheral blood samples and epidemiological data were collected. Plasma isolation occurred within 12 hours of collection and was stored at -80°C for subsequent sequencing.

### Follow-up and data collection

Upon HIV-1 diagnosis, a demographic questionnaire was administered, followed by sample collection, pre-antiviral CD4+T lymphocyte and HIV-1 viral load testing, and initiation of antiviral therapy. Follow-up took place every six months, involving sample collection for CD4+T lymphocyte detection until December 2022.

### RNA extraction and sequencing

Viral RNA extraction from plasma followed the QIAamp Viral RNA Mini Kit protocol (Qiagen, Hilden, Germany). The pol region (HXB2:2147–3462) and env region (HXB2:7002–7663) underwent amplification through nested polymerase chain reaction (Thermo, USA). Sanger sequencing (ThermoFisher Scientific, ABI3500, USA) was performed externally.

### Sequence processing and HIV-1 subtyping

Sequences were edited using Sequencher v5.1 software (Genecodes, Ann Arbor, MI) and aligned using BioEdit 7.1 software (Ibis Biosciences, Carlsbad, CA, USA). Subtyping utilized 117 reference sequences encompassing all Chinese subtypes from the Los Alamos HIV database. Phylogenetic trees were constructed using MEGA 11.0 software employing the neighbor-joining method for subtype identification.

### Receptor tropism prediction

Env sequences were uploaded into GeneCutter HIV (https://www.hiv.lanl.gov/content/sequence/gene_cutter/cutter.html) for V3 region extraction. The V3 region underwent receptor tropism prediction using the geno2pheno website (FRP ≤ 2) and analysis of CXCR4 receptor via the Web PSSM website (https://indra.mullins.microbiol.washingt-on.edu/webpssm/).

### Mutation analysis

Amino acid sequences in the V3 region underwent mutation analysis. Sequences were saved in fasta format and analyzed using the WebLogo website (http://weblogo.berke-ley.edu/logo.cgi).

### Statistical analysis

Demographic information was represented in frequency and percentage. Mann-Whitney U test compared pre-treatment CD4+T lymphocyte counts among different subtypes. Chi-square tests assessed CD4+T lymphocyte distribution pre-treatment. Logistic regression was employed to analyze factors influencing pre-treatment CD4+T lymphocytes, while post-treatment CD4+T lymphocytes recovery utilized LOESS and GEE models. Significance was set at *p* < 0.05 using IBM SPSS 26 for statistical analysis, Python for GEE analysis, and GraphPad Prism 9 for visualization.

## Results

### Characterization of the study population

A total of 1867 individuals were included in the study, including 956 cases involved men who have sex with men (MSM), 836 HET, and 75 cases from other infection routes. In the study population, we enrolled a higher number of HIV-1 infected men (77.03%) compared to women (22.97%). This distribution reflects the actual epidemiological patterns in Guangxi, where HIV-1 prevalence is significantly higher among men. The prevalent HIV-1 subtypes included CRF07_BC (36.48%), CRF01_AE (35.67%), CRF08_BC (12.32%), CRF55_01B (8.25%), and other (7.28%) (**Supplementary Figure 1**). Statistically significant differences were observed among subtypes concerning gender, age, marital status, education, occupation, infection route, infection time, pre-treatment CD4 T cell counts, pre-treatment Viral load, clinical stage, and drug resistance before treatment (*p* < 0.05) (**Table 1**).

**Table 1:** Characterization of the study population among various subtypes.

Variables	CRF01_AE N (%)	CRF07_BC N (%)	CRF08_BC N (%)	CRF55_01B N (%)	Other N (%)	*p* value
Gender						<0.001
Female	153 (22.97)	48 (7.05)	89 (38.7)	2 (1.3)	21 (15.44)	
Male	513 (77.03)	633 (92.95)	141 (61.3)	152 (98.7)	115 (84.56)	
Age						<0.001
< 30	205 (30.78)	392 (57.56)	26 (11.3)	99 (64.29)	66 (48.53)	
30–49	255 (38.29)	208 (30.54)	100 (43.48)	41 (26.62)	47 (34.56)	
≧ 50	206 (30.93)	81 (11.89)	104 (45.22)	14 (9.09)	23 (16.91)	
Ethnicity						0.354
Han	333 (50)	385 (56.53)	122 (53.04)	87 (56.49)	71 (52.21)	
Zhuang	294 (44.14)	262 (38.47)	95 (41.3)	56 (36.36)	60 (44.12)	
Other	39 (5.86)	34 (4.99)	13 (5.65)	11 (7.14)	5 (3.68)	
Marital status						<0.001
Unmarried	288 (43.24)	496 (72.83)	59 (25.65)	120 (77.92)	88 (64.71)	
Married	294 (44.14)	139 (20.41)	134 (58.26)	24 (15.58)	38 (27.94)	
Divorced/widowed	84 (12.61)	46 (6.75)	37 (16.09)	10 (6.49)	10 (7.35)	
Educational level						<0.001
College and above	189 (28.38)	370 (54.33)	13 (5.65)	87 (56.49)	64 (47.06)	
High school or technical school	118 (17.72)	139 (20.41)	23 (10)	39 (25.32)	22 (16.18)	
Junior high school and below	359 (53.9)	172 (25.26)	194 (84.35)	28 (18.18)	50 (36.76)	
Occupation						<0.001
Unemployed	226 (33.93)	175 (25.7)	116 (50.43)	47 (30.52)	40 (29.41)	
Peasant	107 (16.07)	37 (5.43)	48 (20.87)	3 (1.95)	14 (10.29)	
Individual business	62 (9.31)	98 (14.39)	15 (6.52)	21 (13.64)	18 (13.24)	
Student	42 (6.31)	109 (16.01)	2 (0.87)	25 (16.23)	17 (12.5)	
Clerk	76 (11.41)	142 (20.85)	5 (2.17)	24 (15.58)	23 (16.91)	
Workers	66 (9.91)	47 (6.9)	19 (8.26)	19 (12.34)	7 (5.15)	
Services	38 (5.71)	54 (7.93)	8 (3.48)	10 (6.49)	9 (6.62)	
other	49 (7.36)	19 (2.79)	17 (7.39)	5 (3.25)	8 (5.88)	
Infectious route						<0.001
HET	396 (59.46)	175 (25.7)	194 (84.35)	31 (20.13)	40 (29.41)	
MSM	242 (36.34)	498 (73.13)	10 (4.35)	122 (79.22)	84 (61.76)	
Other	28 (4.2)	8 (1.17)	26 (11.3)	1 (0.65)	12 (8.82)	
Infection time						<0.001
Recent	79 (11.86)	168 (24.67)	4 (1.74)	42 (27.27)	26 (19.12)	
Long-term	138 (20.72)	284 (41.7)	6 (2.61)	68 (44.16)	52 (38.24)	
Unknown	449 (67.42)	229 (33.63)	220 (95.65)	44 (28.57)	58 (42.65)	
Pre-treatment CD4 T cell counts, cells/mm						<0.001
< 350	420 (63.06)	319 (46.84)	125 (54.35)	90 (58.44)	59 (43.38)	
≧350	246 (36.94)	362 (53.16)	105 (45.65)	64 (41.56)	77 (56.62)	
Pre-treatment Viral load, copies/ml						<0.001
<10000	17 (2.55)	51 (7.49)	7 (3.04)	5 (3.25)	10 (7.35)	
10000–99999	93 (13.96)	171 (25.11)	22 (9.57)	32 (20.78)	24 (17.65)	
≧100000	65 (9.76)	107 (15.71)	18 (7.83)	39 (25.32)	22 (16.18)	
Unknown	491 (73.72)	352 (51.69)	183 (79.57)	78 (50.65)	80 (58.82)	
Clinical stage						<0.001
I	434 (65.19)	591 (86.81)	141 (61.11)	130 (84.44)	105 (77.39)	
II	62 (9.36)	51 (7.51)	37 (16.11)	13 (8.15)	6 (4.35)	
III	47 (7.07)	13 (1.84)	26 (11.11)	2 (1.48)	8 (6.09)	
IV	122 (18.37)	26 (3.84)	27 (11.67)	9 (5.93)	17 (12.17)	
Drug resistance						<0.001
Yes	42 (6.31)	30 (4.41)	30 (13.04)	11 (7.14)	10 (7.35)	
No	624 (93.69)	651 (95.59)	200 (86.96)	143 (92.86)	126 (92.65)	

Percentage number under parentheses, Chi-square test was used to analyze the difference of DRM frequencies between Han and Zhuang.

### Factors influencing pre-treatment CD4+T lymphocytes and CD4+T lymphocyte recovery

Multivariate logistic regression was utilized to analyze the risk factors affecting pre-Treatment CD4+T lymphocytes. In comparison to factors such as age below 30, Unmarried, Unemployed, other Infection route, and subtypes like CRF07_BC and CRF08_BC, recent infection, and Pre-treatment Viral load <10000 copies/ml, factors associated with a higher risk for pre-treatment CD4+T lymphocyte decline included age over 30, divorced/widowed, peasant, heterosexual infection, CRF01_AE subtype, long-term infection, and pre-treatment viral load >10000 copies/ml. These factors were identified as significant contributors to pre-treatment CD4+T lymphocyte decline (**Supplementary Table 1**).

Generalized Estimation Equation analysis was employed to investigate factors influencing CD4+T lymphocyte recovery. The analysis revealed that infection time, HIV-1 subtype, infection route, gender, age, pre-treatment CD4+T lymphocyte count, clinical stage, treatment regimen, duration time for initiated treatment, and treatment time significantly impacted post-treatment CD4+T lymphocyte recovery. Specifically, male, age over 30, heterosexual infection, long-term infection, CRF01_AE subtype, and pre-treatment CD4 T cell counts < 350/µL, clinical III and IV stage, and longer initiated treatment time were identified as risk factors impeding CD4+T lymphocyte recovery. Interestingly, 3TC+AZT+LPV/r and 3TC+LPV/r+TDF were more conducive to CD4 T cell recovery than 3TC+EFV+TDF, 3TC+AZT+EFV and other. Longer treatment time was beneficial for CD4 T cell recovery (**Table 2**).

**Table 2:** Analysis of factors associated with CD4+T Lymphocytes recovery.

Variables	Multifactor analysis of GEE	
	β, 95%CI	p value
Gender		
Female	Ref	
Male	-58.2 (-86.5, -30.0)	<0.0001
Age		
< 30	Ref	
30–50	-34.6 (-61.2, -8)	0.005
> 50	-77.8 (-112.7, -43)	<0.0001
Ethnicity		
Han	Ref	
Zhuang	-0.1 (-17.3,17.2)	0.511
Other	33.7 (-6.2,73.7)	0.097
Marital status		
Unmarried	Ref	
Married	13.3 (-16,42.7)	0.458
Divorced/widowed	23.7 (-13.4,60.9)	0.486
Educational level		
College and above	Ref	
High school or technical school	20.5 (-4.8,45.9)	0.113
Junior high school and below	-19.2 (-44.4,5.9)	0.133
Occupation		
Unemployed	Ref	
Peasant	-10.7(-41.5,20.1)	0.495
Individual business	36.3(5.4,67.2)	0.021
Student	6(-34.3,46.4)	0.771
Clerk	15.7(-13.9,45.4)	0.299
Workers	-7.8(-43.2,27.6)	0.667
Services	18.3(-26.2,62.9)	0.42
other	-20.4(-74.9,34.1)	0.463
Infected route		
HET	Ref	
MSM	76.0 (33.2,118.7)	<0.0001
Other	26.2 (-46.2,98.6)	0.479
Infection time		
Recent	Ref	
Long-term	-36.0 (-64.8, -7.3)	0.014
Unknown	-0.4 (-44.6,43.7)	0.985
Subtype		
CRF01_AE	Ref	
CRF07_BC	43.2 (20.7,65.8)	<0.0001
CRF08_BC	5.7 (-24.3,35.8)	0.707
CRF55_01B	25.7 (-13.2,64.7)	0.195
Other	46.6 (0.95,92.3)	0.045
Pre-treatment CD4 T cell counts, cells/mm		
< 350	Ref	
≧350	248.6 (229.5,267.7)	<0.0001
Pre-treatment Viral load, copies/ml		
<10000	Ref	
10000–100000	-11 (-47.4,25.4)	0.553
≧100000	-14.8 (-56.5,26.9)	0.486
Unknown	7 (-26.8,40.9)	0.684
Clinical stage		
I	Ref	
II	26.8 (-7.8,58.6)	0.11
III	-61 (-98.8, -24.1)	0.001
IV	-95.9 (-131.0, -60.7)	<0.001
Treatment regimen		
3TC+EFV+TDF	Ref	
3TC+AZT+EFV	24.5 (-7.1,56.2)	0.128
3TC+AZT+LPV/r	67.2 (22.6,111.7)	0.003
3TC+LPV/r+TDF	79 (27.0,131.1)	0.003
Other	-41.2 (-73.0, -9.4)	0.011
Drug resistance		
With PDR	Ref	
Without PDR	-20.6 (-54.7,13.5)	0.234
Duration time for initiated treatment	-1.5 (-2.5,10.5)	0.003
Treatment time	2.7 (2.2,3.2)	<0.0001

GEE model was utilized to analyze these factors associated with CD4+T Lymphocytes recovery, PDR: Pre-treatment drug resistance.

### Effects of subtypes on pre-treatment CD4+T lymphocytes and CD4+T lymphocyte recovery

**Figure 1:**
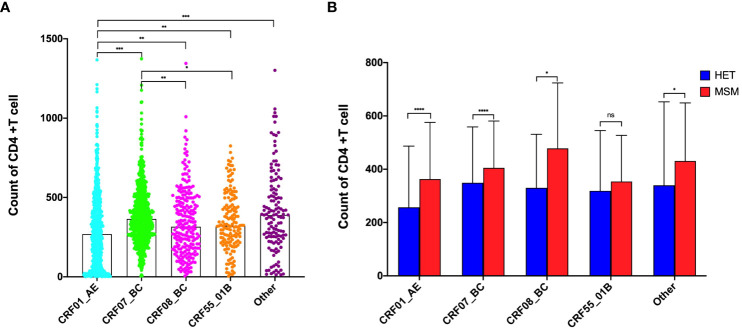
Effects of subtypes on Pre-Treatment CD4+T lymphocytes. **(A)** Effects of subtypes on Pre-Treatment CD4+T lymphocytes among all population; **(B)** Effects of subtypes on Pre-Treatment CD4+T lymphocytes between HET and MSM population. *Represents *P*<0.05, **represents *P*<0.01, ***represents *P*<0.001. ns represents P>0.05.

A comparative analysis was conducted to evaluate the impact of various HIV-1 subtypes on pre-treatment CD4+T lymphocytes. The results revealed significantly lower absolute CD4+T lymphocyte counts among individuals infected with CRF01_AE subtype compared to other subtypes. Conversely, those infected with CRF07_BC subtype demonstrated notably higher CD4+T lymphocyte counts compared to CRF08_BC and CRF55_01B (**Figure 1A**). This trend persisted at the subtype level, where the MSM population demonstrated significantly higher pre-treatment CD4+T lymphocyte counts across CRF01_AE, CRF07_BC, and CRF08_BC subtypes than HET population (*p* < 0.001), with no notable difference in the CRF55_01B subtype (**Figure 1B**).

**Figure 2:**
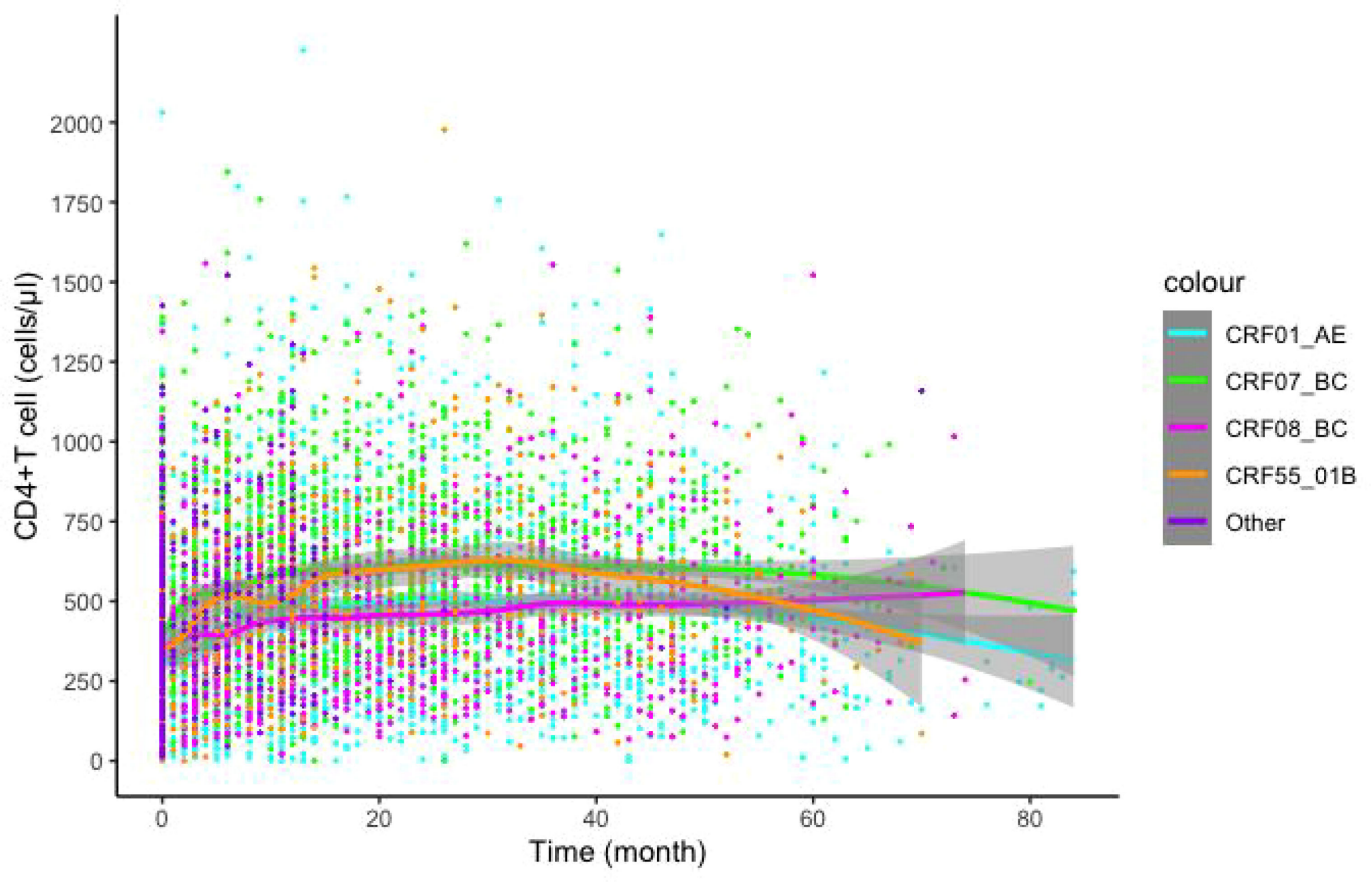
Effects of different subtypes on CD4+T lymphocyte recovery post-Treatment. LOESS model was utilized to predict post-treatment CD4+T lymphocytes recovery, color indicates different subtypes.

The LOESS model was used to fit the recovery trend of CD4 cells after infection with different subtypes. Plotted data included scatter plots and 95% confidence intervals (CI). Higher fitting curves were observed for CRF07_BC and CRF55_01B subtypes compared to CRF01_AE and CRF08_BC subtypes (**Figure 2**). Additionally, we further analyzed the recovery trend of CD4 cells after infection with different subtypes in MSM and HET populations. CRF07_BC infection exhibited a more pronounced recovery effect in the MSM population, while CRF01_AE infection showed comparatively slower recovery. In the HET population, CRF07_BC infection demonstrated the most favorable recovery, followed by CRF08_BC infection, whereas CRF01_AE infection exhibited the least favorable recovery. Intriguingly, the recovery effect of CRF01_AE and CRF07_BC within the MSM population surpassed that observed within the HET population (**Supplementary Figure 2**).

### Effects of subtypes on survival risk

**Figure 3:**
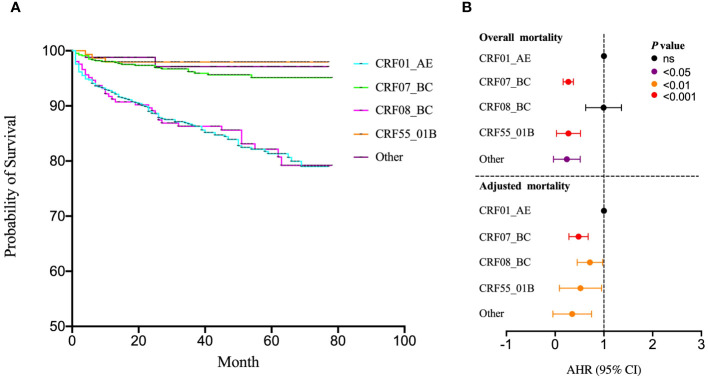
Effects of subtypes on survival risk. **(A)** The COX regression model was utilized to evaluate the influence of various HIV-1 subtypes on survival risk; **(B)** The univariate COX regression was used to analysis the survival risk across subtypes incorporating gender, age, marital status, education, occupation, infection route, pre-treatment CD4 T cell counts, pre-treatment Viral load, clinical stage, drug resistance, treatment regimen, duration time for initiated treatment and treatment. ns represents P>0.05.

The COX regression model was utilized to evaluate the influence of various HIV-1 subtypes on survival risk. The survival curve unveiled indicated higher mortality rates for CRF01_AE and CRF08_BC compared to CRF07_BC, CRF55_01B and other subtypes (**Figure 3A**). Using CRF01_AE as the reference, the univariate COX regression demonstrated significant differences in hazard ratios (HR), notably highlighting CRF07_BC (HR=0.26), CRF55_01B (HR=0.19) and other subtypes (HR=0.14). Furthermore, adjusted through gender, age, marital status, education, occupation, infection route, pre-treatment CD4 T cell counts, pre-treatment Viral load, clinical stage, drug resistance, treatment regimen, duration time for initiated treatment, and treatment time, the result revealed significant differences between CRF01_AE (reference), CRF07_BC (AHR=0.46), CRF08_BC (AHR=0.68), CRF55_01B (AHR=0.4), and other subtypes (AHR=0.2) (**Figure 3B**).

### Prediction of receptors selection and disease progression among subtypes

**Figure 4:**
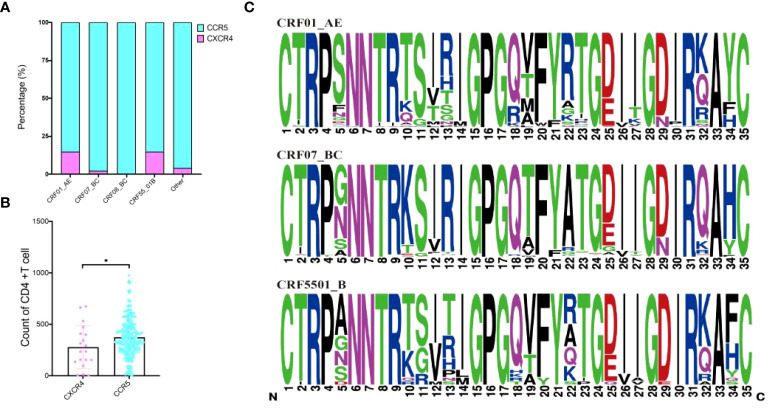
Prediction of receptors selection and mutation within CRF01_AE, CRF07_BC and CRF55_01B. **(A)** Prediction of receptors selection in various subtypes; **(B)** Comparative analysis of CD4+T cell counts between CXCR4 and CCR5 group; **(C)** Comparative analysis of V3 region sequences within CRF01_AE, CRF07_BC and CRF55_01B. *Represents P<0.05.

To explore the relationship between various HIV-1 subtypes and the selection of CXCR4 and CCR5 receptor, sequencing focused on the membrane protein receptor (env) region was conducted, successfully sequencing 281 cases. The analysis revealed that among the sequenced samples, 19 were associated with CXCR4 tropism, while the remaining cases indicated either CCR5 tropism or a mixed tropic affinity. When stratified by subtypes, the CXCR4 affinity ratios were distributed as follows: CRF01_AE subtype exhibited 14.67% (11/75), CRF55_01B subtype displayed 14.81% (4/27), CRF07_BC subtype indicated 2.19% (3/137), and CRF08_BC subtype showed 0.00%. Other subtypes accounted for 4.00% (1/25) (**Figure 4A**). Furthermore, significant differences in CD4+T cell counts were observed between individuals exhibiting CXCR4 and CCR5 receptor affinity (**Figure 4B**). Comparative analysis of V3 region sequences within CRF01_AE, CRF07_BC and CRF55_01B unveiled significant mutation at positions 5, 10, 11, 12, 13, 18, 19, 22, 25, 29, 32, and 34 (**Figure 4C**). The CD4+ T cell counts were significantly different between mutation S11R, I12T, V12T, T19V, A22R, and N29D (**Supplementary Figure 3**).

## Discussion

Our study aimed to delve into the correlation between HIV-1 genetic diversity and disease progression by examining CD4+T lymphocyte counts, and longitudinal data obtained from HIV-1 patients over various timeframes. We concurrently sequenced HIV-1 env genes in this cohort and utilized online tools to predict CXCR4 and CCR5 receptor affinity. Our findings indicate associations between different subtypes and pre-treatment CD4+T lymphocyte counts, CD4+T lymphocyte recovery, and mortality. These results suggest potential influences of various subtypes on immune status and the receptor tropism of HIV-1-infected individuals.

Notably, infection with the CRF01_AE subtype displayed lower pre-treatment CD4+T lymphocyte counts, likely attributable to its higher CXCR4 receptor ratio. This heightened tropism might contribute to rapid CD4+T lymphocyte decline and accelerated disease progression (Koot et al., 1993). Originating in Central Africa, CRF01_AE is now endemic in Southeast and East Asia. Studies have linked this subtype to a high proportion of CXCR4-tropic virus, hastening AIDS development and immune failure (Kaufmann et al., 2005; Kelley et al., 2009). However, our research indicates that the CXCR4 receptor tropism ratio in Guangxi population is lower compared to other regions (Li et al., 2016; Cui et al., 2019), suggesting additional factors affecting CD4+T lymphocyte counts. Factors such as advanced age, peasant, heterosexual infection, long-term infection, and higher viral load significantly impact CD4+T lymphocytes. Previous studies (Ge et al., 2019) in Guangxi reported a higher proportion of individuals among age over 50, peasant, and heterosexual infection, contributing to low pre-treatment CD4 T lymphocyte counts, which affect treatment efficacy. Additionally, patients with the CRF07_BC subtype exhibited improved CD4+T lymphocyte recovery, consistent with prior literature (Jiang et al., 2016). We hypothesize that compared to other subtypes, CRF07_BC infection might delay CD4+T lymphocyte decline and clinical progression in HIV-1 patients, potentially extending survival time and increasing transmission risks. The study also revealed disparities in CD4+T lymphocyte recovery between MSM and heterosexual populations infected with the CRF07_BC subtype, potentially contributing to the subtype’s rapid increase in the Guangxi MSM population. One potential hypothesis is that the CRF07_BC subtype may be associated with a less aggressive disease course, allowing for better immune system preservation, especially in populations with early and consistent access to healthcare. The higher pre-treatment CD4+T lymphocyte counts in MSM populations could also be attributed to earlier diagnosis and initiation of ART, reflecting the importance of timely medical intervention in managing HIV infection. In conclusion, the disparities observed in our study highlight the need for further research to understand the underlying mechanisms driving these differences. Future studies should investigate the role of viral genetic factors, host immune responses, and sociocultural influences in shaping the disease course of different HIV-1 subtypes in diverse populations. This understanding can inform targeted interventions and improve health outcomes for all individuals living with HIV.

Moreover, employing the 11/25 rule, which correlates amino acid residues at positions 11 or 25 of the V3 region with CXCR4 receptor tropism (Sander et al., 2007), we observed relatively low amino acid substitutions in the Guangxi population. Specifically, 11R accounted for 21.1% (4/19) of CXCR4 receptor tropism, primarily within the CRF55_01B subtype. Furthermore, mutations at sites 11 and 25 were found in CRF07_BC and CRF59_01B subtypes, indicating diverse evolutionary pathways for tropism conversion. Studies from France have highlighted multiple amino acid residue substitutions, including S5Y, N7K, S11R, T12V, T12F, Q18R, I27T, and S32R in the V3 region of the CRF01_AE subtype. The substitution of arginine (R) for serine (S) at site 11 is pivotal for CCR5 tropic strains’ transformation into CXCR4 tropic strains (Shoombuatong et al., 2012; Hongjaisee et al., 2017). Our study identified mutations like S11R, I12T, V12T, T19V, A22R, and N29D as key sites leading to CD4+T cell number decline. These mutations were pivotal for the transformation from CCR5 to CXCR4 tropism is particularly noteworthy. Notably, 12T (46.7%) and 11R (100%) exhibited higher CXCR4 tropism. Notably, the CRF01_AE subtype exhibited a higher proportion of CXCR4-tropic viruses, potentially contributing to a more rapid decline in CD4+T lymphocytes and accelerated disease progression. However, the S11R mutation in our study was mainly concentrated in CRF55_01B, which is why the CRF55_01B subtype displayed 14.81% CXCR4 affinity ratios. This indicates that the S11R mutation is not only the specific site of CRF01_AE subtype, but also that the high proportion of CXCR4 in the CRF01_AE subtype in Guangxi is not due to the S11R mutation but may be affected by other mutation. For example, I12T, V12T, T19V, 273, A22R, and N29D. Which mutation affects CRF01_AE subtype tropism selection, whether it is single mutation or double mutation, needs to be further studied. This reflects that the mutation affecting the tropism selection of subtype in different regions are different. This subtype-specific characteristic could be a significant factor in understanding the disease dynamics in the Guangxi region. However, there is a recognized lack of consensus regarding the necessary substitutions for CXCR4 tropism in this subtype (Hongjaisee et al., 2017). This discrepancy highlights the necessity for biological evaluation of coreceptor tropism of these variants to confirm tropism. Our research sheds light on the relationship between HIV-1 V3 region sequence variation and coreceptor preference in Guangxi’s populations, identifying characteristic mutations potentially linked to the geographical environment.

While our study provides valuable insights into the correlation between HIV-1 genetic diversity and disease progression in Guangxi, it is imperative to acknowledge certain limitations. Firstly, our research predominantly focuses on the prevalent subtypes in Guangxi, namely CRF01_AE, CRF07_BC, CRF08_BC, and CRF55_01B. The exclusion of less common subtypes may limit the overall representativeness of our results. Additionally, the collected data may introduce inherent biases and potential inaccuracies in patient histories. For example, women were significantly underrepresented in the sample, reflecting the higher prevalence of HIV-1 among men in Guangxi. This gender disparity may limit the generalizability of our conclusions to the female population. Furthermore, the predictive analysis of receptor tropism, while informative, is based on computational tools and sequencing data, which may not fully capture the *in vivo* complexity of viral dynamics. Lastly, the study’s scope is limited to Guangxi, and caution should be exercised when extrapolating these findings to other regions with distinct epidemiological profiles. Despite these limitations, our research contributes valuable insights to the existing body of knowledge and lays the groundwork for future investigations in this critical area of HIV research.

## Conclusion

Our study emphasizes the association between pre-treatment CD4+T lymphocyte counts, recovery, survival risk, and HIV-1 gene diversity in Guangxi. Lower CD4+T lymphocyte counts and slower recovery in CRF01_AE infected individuals contrast with higher counts and faster recovery in CRF07_BC infected individuals, potentially contributing to the increased AIDS prevalence and rapid CRF07_BC expansion in Guangxi. Multiple influencing factors and distinct mutation further impact CD4+T lymphocyte counts and recovery in HIV-infected individuals in Guangxi.

## Data availability statement

The datasets presented in this study can be found in online repositories. The names of the repository/repositories and accession number(s) can be found in the article/**Supplementary Material**.

## Ethics statement

The studies involving humans were approved by Ethics Review Board of Guangxi Center for Disease Control and Prevention. The studies were conducted in accordance with the local legislation and institutional requirements. The participants provided their written informed consent to participate in this study. Written informed consent was obtained from the individual(s) for the publication of any potentially identifiable images or data included in this article.

## Author contributions

SL: Writing – review & editing, Supervision, Project administration, Funding acquisition, Conceptualization. XP: Writing – original draft, Visualization, Validation, Software, Resources, Methodology, Investigation, Formal analysis, Data curation. JH: Writing – review & editing, Validation, Software, Data curation. KT: Writing – review & editing, Validation, Investigation, Data curation. JM: Writing – review & editing, Validation, Investigation, Data curation. NF: Writing – review & editing, Validation, Methodology, Data curation. HX: Writing – review & editing, Resources, Data curation. QH: Writing – review & editing, Methodology, Data curation. QZ: Writing – review & editing, Validation, Supervision, Project administration. GL: Writing – review & editing, Supervision, Project administration, Funding acquisition, Conceptualization.
