# Supplementary material for: Impact of HIV-1 genetic diversity on disease progression: a prospective cohort study in Guangxi
Source: Front Cell Infect Microbiol. 2024 Jun 27;14:1415123. doi: 10.3389/fcimb.2024.1415123 (PMC11236547; doi:10.3389/fcimb.2024.1415123)
Supplement: Supplementary file 1 [file DataSheet_1.docx]

**Supplementary material**

**Figure S1 The phylogenetic trees for subtype identification**

**
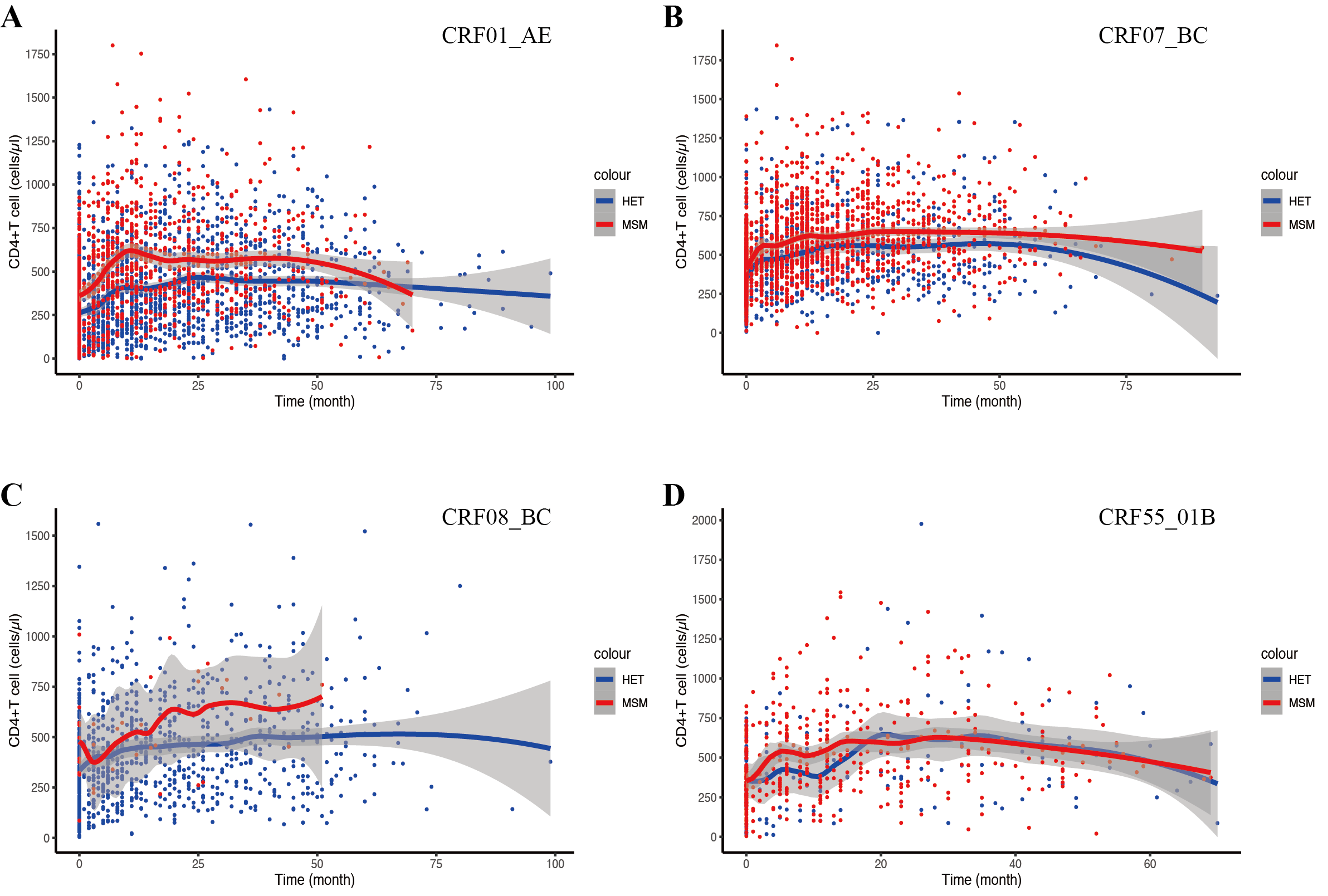
**

**Figure S2** Effects of different subtypes on CD4+T lymphocyte recovery across MSM and HET population. A: CRF01_AE; B:CRF07_BC; CRF08_BC; CRF55_01B.


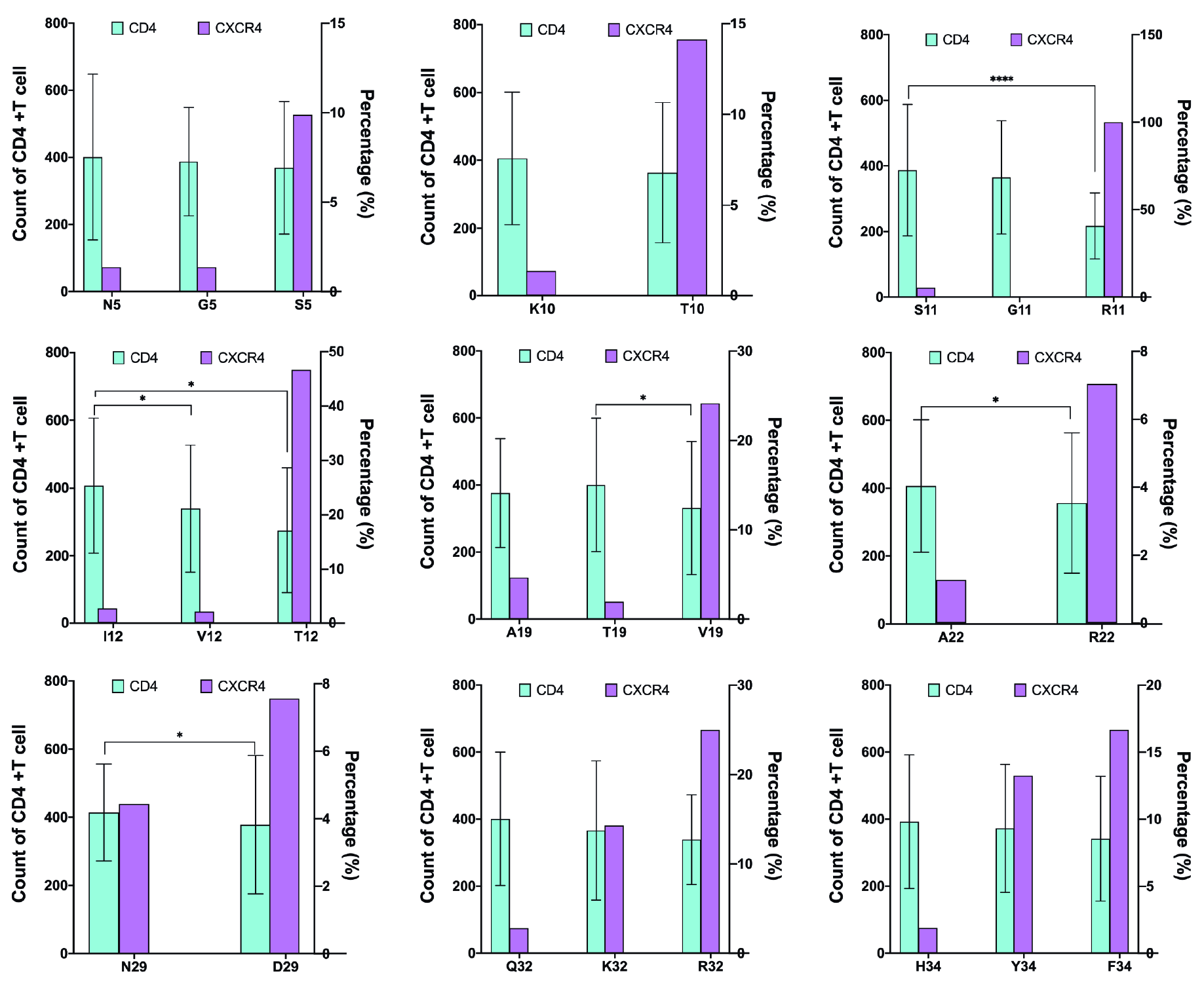


**Figure S3** Correlation analysis between mutation and pre-treatment CD4+T cell. *represents p<0.05, **represents p<0.01, ***represents p<0.001.

**Table S1** Analysis of influencing factor associated with Pre-treatment CD4+ T lymphocyte counts

| Variables | Pre-treatment CD4 T cell counts, cells/mm | | OR (95% CI) | |
| --- | --- | --- | --- | --- |
|  | <350 | ≧350 | Unadjusted | Adjusted |
| Gender |  |  |  |  |
| Female | 184 | 129 | Ref |  |
| Male | 832 | 722 | 1.24 (0.97-1.58) |  |
| Age |  |  |  |  |
| < 30 | 347 | 441 | Ref | Ref |
| 30-49 | 372 | 279 | 0.59 (0.48-0.73)*** | 0.75 (0.56-1)* |
| ≧ 50 | 297 | 131 | 0.35 (0.27-0.45)*** | 0.61 (0.41-0.91)* |
| Ethnicity |  |  |  |  |
| Han | 545 | 456 | Ref |  |
| Zhuang | 415 | 352 | 1.01 (0.84-1.22) |  |
| Other | 56 | 43 | 0.92 (0.61-1.39) |  |
| Marital status |  |  |  |  |
| Unmarried | 485 | 566 | Ref | Ref |
| Married | 403 | 226 | 0.48 (0.39-0.59)*** | 0.82 (0.59-1.13) |
| Divorced/widowed | 128 | 59 | 0.4 (0.28-0.55)*** | 0.65 (0.43-1)* |
| Educational level |  |  |  |  |
| College and above | 355 | 368 | Ref |  |
| High school or technical school | 172 | 169 | 0.95 (0.73-1.23) | 1.2 (0.9-1.61) |
| Junior high school and below | 489 | 314 | 0.62 (0.51-0.76)*** | 1.08 (0.8-1.47) |
| Occupation |  |  |  |  |
| Unemployed | 313 | 291 | Ref | Ref |
| Peasant | 149 | 60 | 0.43 (0.31-0.61)*** | 0.65 (0.45-0.95)* |
| Individual business | 105 | 109 | 1.12 (0.82-1.53) | 1.09 (0.78-1.53) |
| Student | 80 | 115 | 1.55 (1.12-2.14)** | 1.1 (0.75-1.61) |
| Clerk | 143 | 127 | 0.96 (0.72-1.27) | 0.82 (0.59-1.14) |
| Workers | 94 | 64 | 0.73 (0.51-1.05) | 0.8 (0.55-1.16) |
| Services | 60 | 59 | 1.06 (0.71-1.57) | 1.03 (0.67-1.57) |
| Other | 72 | 26 | 0.39 (0.24-0.63)*** | 0.55 (0.33-0.91) |
| Infectious route |  |  |  |  |
| HET | 540 | 296 | Ref | Ref |
| MSM | 445 | 511 | 2.1 (1.73-2.53)*** | 1.37 (0.84-2.24) |
| Other | 31 | 44 | 2.59 (1.6-4.19)*** | 1.85 (1.09-3.13)* |
| Subtype |  |  |  |  |
| CRF01_AE | 420 | 246 | Ref |  |
| CRF07_BC | 319 | 362 | 1.94 (1.56-2.41)*** | 1.53 (1.2-1.95)** |
| CRF08_BC | 128 | 102 | 1.36 (1-1.84)* | 1.66 (1.2-2.32)** |
| CRF55_01B | 90 | 64 | 1.21 (0.85-1.74) | 0.95 (0.65-1.4) |
| Other | 59 | 77 | 2.23 (1.53-3.24)*** | 1.87 (1.25-2.78)** |
| Infection time |  |  |  |  |
| Recent | 124 | 195 | Ref | Ref |
| Long-term | 278 | 270 | 0.62 (0.47-0.82)*** | 0.48 (0.35-0.65)*** |
| Unknow | 614 | 386 | 0.4 (0.31-0.52)*** | 0.51 (0.31-0.84)** |
| Pre-treatment Viral load, copies/ml |  |  |  |  |
| <10000 | 32 | 66 | Ref | Ref |
| 10000-99999 | 172 | 168 | 0.47 (0.3-0.76)** | 0.49 (0.3-0.81)** |
| ≧100000 | 168 | 85 | 0.25 (0.15-0.4)*** | 0.24 (0.14-0.4)*** |
| Unknow | 644 | 532 | 0.4 (0.26-0.62)*** | 0.67 (0.42-1.07) |
| If drug resistance |  |  |  |  |
| Yes | 72 | 51 | Ref |  |
| No | 944 | 800 | 0.84 (0.58-1.21) |  |

Logistic regression was employed to analyze factors influencing pre-treatment CD4+T lymphocytes, *represents *p*<0.05, **represents *p*<0.01, ***represents *p*<0.001.
